# Melanopsin Ganglion Cells Are the Most Resistant Retinal Ganglion Cell Type to Axonal Injury in the Rat Retina

**DOI:** 10.1371/journal.pone.0093274

**Published:** 2014-03-26

**Authors:** Luis Pérez de Sevilla Müller, Allison Sargoy, Allen R. Rodriguez, Nicholas C. Brecha

**Affiliations:** 1 Department of Neurobiology, David Geffen School of Medicine at the University of California Los Angeles, Los Angeles, California, United States of America; 2 Department of Medicine, David Geffen School of Medicine at the University of California Los Angeles, Los Angeles, California, United States of America; 3 Jules Stein Eye Institute, David Geffen School of Medicine at the University of California Los Angeles, Los Angeles, California, United States of America; 4 CURE Digestive Diseases Research Center, David Geffen School of Medicine at the University of California Los Angeles, Los Angeles, California, United States of America; 5 Veterans Administration Greater Los Angeles Health System, Los Angeles, California, United States of America; Morehouse School of Medicine, United States of America

## Abstract

We report that the most common retinal ganglion cell type that remains after optic nerve transection is the M1 melanopsin ganglion cell. M1 ganglion cells are members of the intrinsically photosensitive retinal ganglion cell population that mediates non-image-forming vision, comprising ∼2.5% of all ganglion cells in the rat retina. In the present study, M1 ganglion cells comprised 1.7±1%, 28±14%, 55±13% and 82±8% of the surviving ganglion cells 7, 14, 21 and 60 days after optic nerve transection, respectively. Average M1 ganglion cell somal diameter and overall morphological appearance remained unchanged in non-injured and injured retinas, suggesting a lack of injury-induced degeneration. Average M1 dendritic field size increased at 7 and 60 days following optic nerve transection, while average dendritic field size remained similar in non-injured retinas and in retinas at 14 and 21 days after optic nerve transection. These findings demonstrate that M1 ganglion cells are more resistant to injury than other ganglion cell types following optic nerve injury, and provide an opportunity to develop pharmacological or genetic therapeutic approaches to mitigate ganglion cell death and save vision following optic nerve injury.

## Introduction

Retinal, optic nerve and brain injury may lead to vision loss by compression or trauma to retinal ganglion cell (RGC) axons that often lead to RGC death. Glaucoma, the second leading cause of blindness worldwide affecting nearly 70 million people [1], as well as optic nerve stroke, cause blindness through nerve injury.

In the retina, more than 50% of RGCs degenerate one week after axotomy [2], [3] and more than 90% of RGCs are lost by the third week after axotomy [2]–[6]. A small percentage of RGCs survive up to one year following axotomy [5]–[7]. The goal of these studies is to identify and characterize the RGCs that survive after optic nerve transection (ONT), and to determine whether they are representative of all RGC types or a subpopulation of RGCs in the rat retina. Knowledge of surviving RGC type morphology and neurochemistry may provide insights into intrinsic RGC protective features that mediate cell survival. These properties could provide the basis for the development of neuroprotective interventions to save vision.

In the present study we have identified and analyzed the RGCs that survive after ONT in the rat retina. We have found that M1 ganglion cells are the most common ganglion cell type that remains in the retina 60 days following optic nerve axotomy, comprising 82±8% of all surviving RGCs.

## Materials and Methods

### Animals

Male adult Sprague-Dawley rats (250–300 g., >1 month old, Charles River Laboratories, Wilmington, MA) were used for these studies. The UCLA Chancellor’s Animal Research Committee has approved the animal care and use protocols (ARC #1998–064) and all of these studies were performed in accordance with ARVO’s Use of Animals in Ophthalmic and Visual Research and PHS Policy on Humane Care and Use of Laboratory Animals. All rat work was performed in accordance with IACUC guidelines.

### Optic Nerve Transection Model

Rats were anesthetized with 3–5% isoflurane in oxygen (1.5 L/min) during ONT. A small incision was made in the temporal conjunctiva of the left eye and gently peeled back posteriorly to avoid cutting blood vessels. The optic nerve sheath was incised 2 mm longitudinally, starting about 2 mm behind the globe to expose the optic nerve. The optic nerve was transected completely by a needle knife without damaging the adjacent blood supply. Direct ophthalmoscopic inspection confirmed there was no bleeding from retinal blood vessels. The right eye was left unoperated and used as a control.

Animals were deeply anesthetized with isoflurane (IsoFlo, Abbott Laboratories) and euthanized by decapitation at 7, 14, 21 or 60 days after axotomy. In rat retina, ONT results in ∼50% loss of RGCs in the ganglion cell layer (GCL) at 7 days and ∼95% loss of cells at 3 weeks after transection, respectively [2], [3].

### Immunohistochemistry

Immunohistochemistry was performed on whole-mount retinas. Antibodies to neurofilament-M (1∶1000, MAB-1621; Millipore, Billerica, MA), melanopsin (1∶250, PA1-781; Thermo Scientific, Waltham, MA) and RNA binding protein with multiple splicing (RBPMS, 1∶1000) were used. The RBPMS polyclonal antibodies were generated against the N-terminus of the RBPMS polypeptide, GGKAEKENTPSEANLQEEEVR, in guinea pig by a commercial vendor (ProSci, Poway, CA), and affinity purified and characterized in our laboratory [8]. Retinas were mounted on cellulose filter paper (Millipore) with the GCL up and fixed in 4% PFA for 10 minutes. Whole-mounted retinas were incubated in 10% normal goat serum at 4°C overnight. The retinas were subsequently incubated in primary antibody for 5–7 days at 4°C, washed three times in phosphate buffer (PB) 0.1 M pH = 7.4 and then incubated overnight at 4°C in the appropriate secondary antibody (1∶500, coupled to Alexa Fluor 488, 633 or Cy3, Invitrogen, Carlsbad, CA). After three final washes in PB, the retinas were mounted in Vectashield Mounting Medium (Vector Laboratories, Burlingame, CA). Coverslips were sealed with nail polish for prolonged storage. Slides were stored at 4°C and protected from light.

### Image Analysis

Images were acquired using a Zeiss Laser Scanning Microscope 510 Meta or 710 (Carl Zeiss, Thornwood, NY) with a Zeiss Plan-Neofluar 25×/0.80 mm or a Zeiss C-Apochromat 40× 1.2 NA corrected water objective at a resolution of 1024 × 1024 pixels. Images are presented as projections consisting of 6–17 optical sections (z-axis step size 0.3–1 μm). Three morphological parameters were analyzed: dendritic field area, dendritic field diameter and somal diameter. Dendritic field area was calculated by using the public domain Java image processing software ImageJ (NIH). Confocal images, dendritic field and somal diameters were analyzed using the Zeiss LSM 510 proprietary software (version 3.2). The intensity levels and contrast of the final images were adjusted in Adobe Photoshop CS2 v.9.02 (Adobe Systems, San Jose, CA).

### Quantification of RGCs

Confocal images were taken at 40x magnification of RBPMS-, NF- and melanopsin immunoreactive cells from whole mounted retinas. Images were collected at 0.5-mm intervals from the optic nerve head to peripheral retina. At least 3 retinal fields per quadrant of each retina were analyzed. Cells were manually counted.

### Statistical Analysis

All values are given as mean ± standard deviation and were compared for statistical difference by using the unpaired Student’s *t*-test. One-way ANOVA followed by Tukey test was used when more than two groups were compared (SigmaPlot; Systat Software Inc., San Jose, CA). P≤0.05 was considered to be statistically significant.

## Results

**Figure 1:**
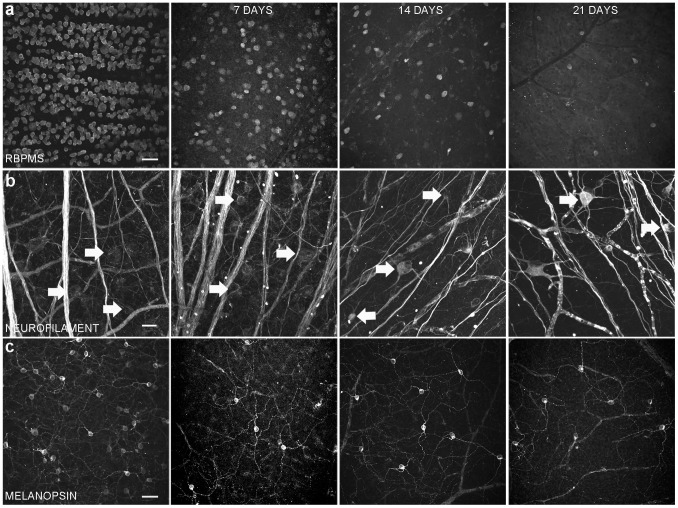
Whole-mounted rat retinas showing the effect of axotomy on RGC number and morphology. Left column is a control retina from the non-operated eye (a) RBPMS immunostaining (a specific marker for ganglion cells) in the ganglion cell layer at 7, 14 and 21 days after optic nerve transection. (b) Neurofilament-M immunostaining (arrows) at 7, 14 and 21 days after optic nerve transection. (c) M1 ganglion cells at 7, 14 and 21 days after optic nerve transection. (a, c) Scale bar = 50****μm; (b) Scale bar = 20 μm.

RGCs remaining after axotomy were identified by RBPMS immunoreactivity (**Fig. 1a**), a selective marker of mammalian RGCs that is primarily expressed in the cell body [8], [9] and neurofilament-M (NF, **Fig. 1b**, arrows), a general marker of large and small RGCs [10]–[14].

### Neurofilament-immunoreactive Cells

**Figure 2:**
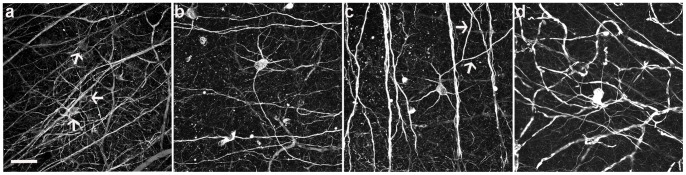
ON α-like ganglion cells are the most resistant type of the neurofilament-M immunoreactive cells after axotomy. (a) Control eye showing ON α-like ganglion cells (arrows) and other neurofilament-M positive ganglion cells in the peripheral area. (b, c) Examples of morphological changes (arrows in C) 21 days after axotomy in the peripheral area. (d) Example of an ON α-like ganglion cell 60 days after optic nerve transection in the peripheral area. Scale bar = 50****μm.

Among the NF-immunoreactive cells, we identified an ON-cell type, based on the distribution of their dendrites in the ON sublamina of the inner plexiform layer (IPL), that survived axotomy (**Fig. 2**). These cells were characterized by three to five stout primary dendrites that branched laterally, and formed a large dendritic field. These cells are among the largest RGCs with a somal diameter of 24.6±3.4 μm (n = 9 cells). The stratification and general morphology of these cells resemble ON α-RGCs in the rat retina [15] (**Fig. 2a** **,** arrows). These cells undergo morphological changes after axotomy, including more than a 50% decrease in their dendritic field (**Fig. 2b**) 21 days after axotomy. In addition, some of these large cells had asymmetric dendritic fields with enlarged dendrites (**Fig. 2c**, arrows), indicative of neuronal damage [16], [17]. Two months after ONT **(** **Fig. 2d** **)**, the ON α-like ganglion cells comprised the majority of NF-positive cells (98±3%, n = 3 retinas).

#### Percentage of surviving NF-immunostained ganglion cells

**Figure 3:**
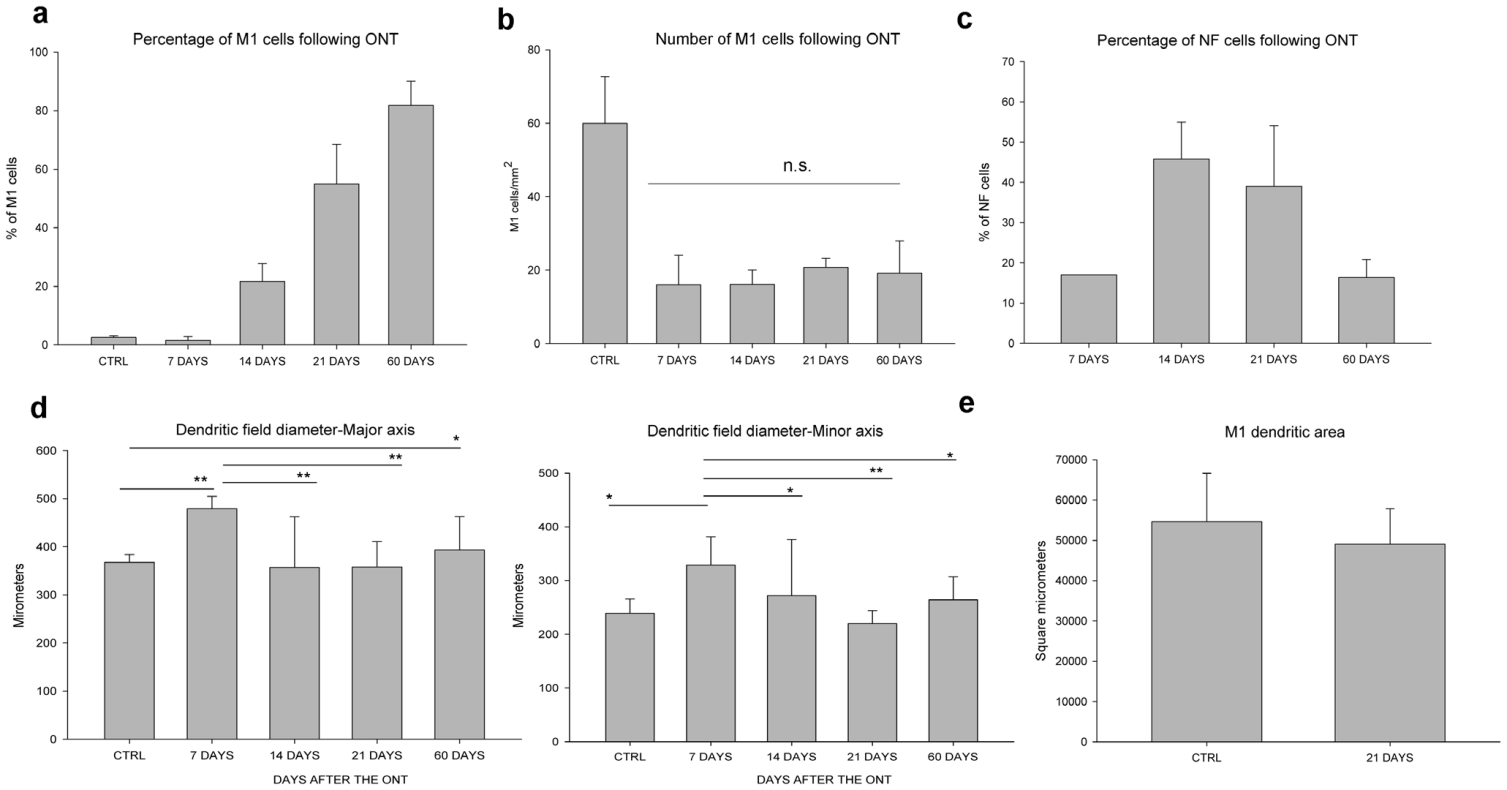
Characterization of surviving M1 and neurofilament-M ganglion cells. Surviving M1 (a) and neurofilament-M ganglion cells (c) compared to the number of RBPMS immunoreactive cells after axotomy. (b) Number of M1 ganglion cells/mm^2^. (d) Dendritic field diameter measurements and (e) Dendritic area of M1 ganglion cells remain unchanged 21 days after axotomy. n.s = no significance, * indicates P<0.05 and ** P<0.001.

The NF-immunostained cells (**Fig. 3c**) constituted 17% of all RBPMS immunoreactive cells (143 NF ganglion cells/mm^2^; n = 1 retina), 46±9% (103±28 NF ganglion cells/mm^2^; n = 4 retinas), 39±15% (21±11 NF ganglion cells/mm^2^; n = 3 retinas), and 16±5% (4±3 NF ganglion cells/mm^2^; n = 3 retinas) of the surviving RGCs 7, 14, 21 and 60 days after ONT, respectively.

### M1 Ganglion Cells

A different subpopulation of RGCs, M1 ganglion cells, which are a component of the intrinsically photosensitive RGC (ipRGC) population that mediates non-image-forming vision [18], were identified using a specific melanopsin antibody [18]–[20]. A large number of the M1 ganglion cells remained after axotomy (**Fig. 1c**).

#### Percentage of surviving M1 ganglion cells

M1 ganglion cells comprise ∼2.5% of all RGCs in the rat retina [18]. In this study, we found M1 ganglion cells comprised 1.7±1% (16±8 M1 ganglion cells of 1065±525 RGCs/mm^2^; n = 3 retinas), 28±14% (29±22 M1 ganglion cells of 156±77 RGCs/mm^2^; n = 4 retinas), 55±13% (21±3 M1 ganglion cells of 46±14 RGCs/mm^2^; n = 3 retinas) and 82±8% (30±7 M1 ganglion cells of 34±4 RGCs/mm^2^; n = 5 retinas) of the surviving RGCs 7, 14, 21 and 60 days after ONT, respectively (**Fig. 3a**). There was an initial decrease of M1 ganglion cell density 7 days after ONT (**Fig. 3b**), while the cell density stabilized at 14, 21 and 60 days after ONT.

#### Soma size

A previous study reported that the average RGC somal diameters reduced 7 days after ONT in the rat retina [21]. In contrast, M1 ganglion cell morphology appeared to be similar in non-injured and ONT retinas; the major somal diameters of M1 ganglion cells from ONT retinas were unchanged (not shown; n = 16 cells from 3 retinas at 7 days after ONT, one-way ANOVA followed by Tukey test p = 0.865; n = 19 cells from 2 retinas at 14 days after ONT, p = 0.960; n = 37 cells from 5 retinas at 21 days after ONT, p = 0.999; n = 28 cells from 3 retinas at 60 days after ONT, p = 0.879) compared to M1 ganglion cells from non-injured retinas (n = 26 M1 ganglion cells from 3 retinas). Similarly the minor somal diameters of M1 ganglion cells from ONT retinas were unchanged (not shown; n = 16 cells from 3 retinas at 7 days after ONT, one-way ANOVA followed by Tukey test p = 0.985; n = 19 cells from 2 retinas at 14 days after ONT, p = 0.498; n = 37 cells from 5 retinas at 21 days after ONT, p = 0.834; n = 28 cells from 3 retinas at 60 days after ONT, p = 0.881) compared to M1 ganglion cells from non-injured retinas (n = 26 M1 ganglion cells from 3 retinas).

#### Dendritic field size

M1 ganglion cell dendritic field size significantly increased 7 days after ONT (n = 16 cells; major dendritic field; one-way ANOVA followed by Tukey test p<0.001; minor dendritic field p = 0.021), yet remained similar in size to M1 ganglion cells in normal retinas and in retinas 14 and 21 days after ONT (**Fig. 3d**; one-way ANOVA followed by Tukey test major dendritic field p = 0.654, p = 0.980, respectively; minor dendritic field p = 1.000, and p = 0.929; respectively). However, the dendritic coverage of M1 ganglion cells was unchanged 21 days after axotomy (**Fig. 3e**; t-test p = 0.633). Interestingly, we observed an increase in the major axis of the dendritic field but not in the minor axis of the dendritic field at 60 days after ONT (n = 21 cells; one-way ANOVA followed by Tukey test, major dendritic field p = 0.038; minor dendritic field p = 0.680).

#### Stratification patterns

**Figure 4:**
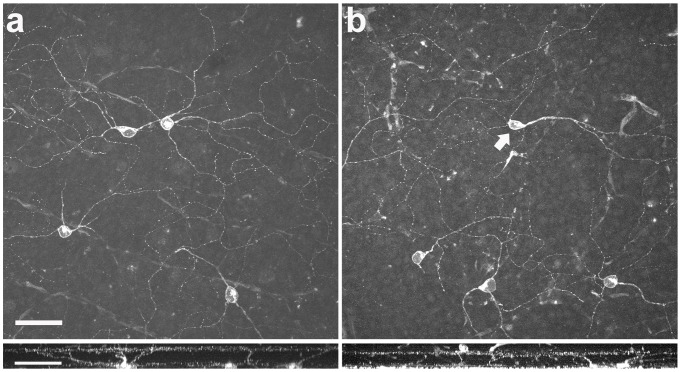
Morphological comparison of M1 ganglion cells in normal and optic nerve transected retina. (a) M1 ganglion cell immunostaining in the control eye. Bottom panel shows a side view with the typical stratification in the OFF sublamina of the inner plexiform layer. (b) M1 ganglion cell immunostaining 60 days after optic nerve transection. A displaced M1 ganglion cell is included (arrow). Bottom panel shows that in the injured retina the M1 ganglion cell dendrites remain in the same lamina of the IPL as in the control retina. Scale bar = 50 μm.

The laminar position of the M1 ganglion cell dendrites in the IPL did not change after axotomy. **Fig. 4a** shows a confocal projection of M1 ganglion cells in a non-injured retina and its ramification in lamina 1 of the IPL. Sixty days after the ONT, M1 ganglion cell dendrites ramified in the same lamina of the IPL (**Fig. 4b**).

### Other Surviving RGCs

In addition to M1 and ON α-like ganglion cells, triple labeling experiments showed 1.2±0.3% of the RGCs identified by RBPMS immunoreactivity as neither M1- nor NF-positive in the GCL 60 days after surgery with a soma size of 9.8±3.7 μm (n = 5 cells from 2 retinas; see Discussion).

### Displaced RGCs

**Figure 5:**
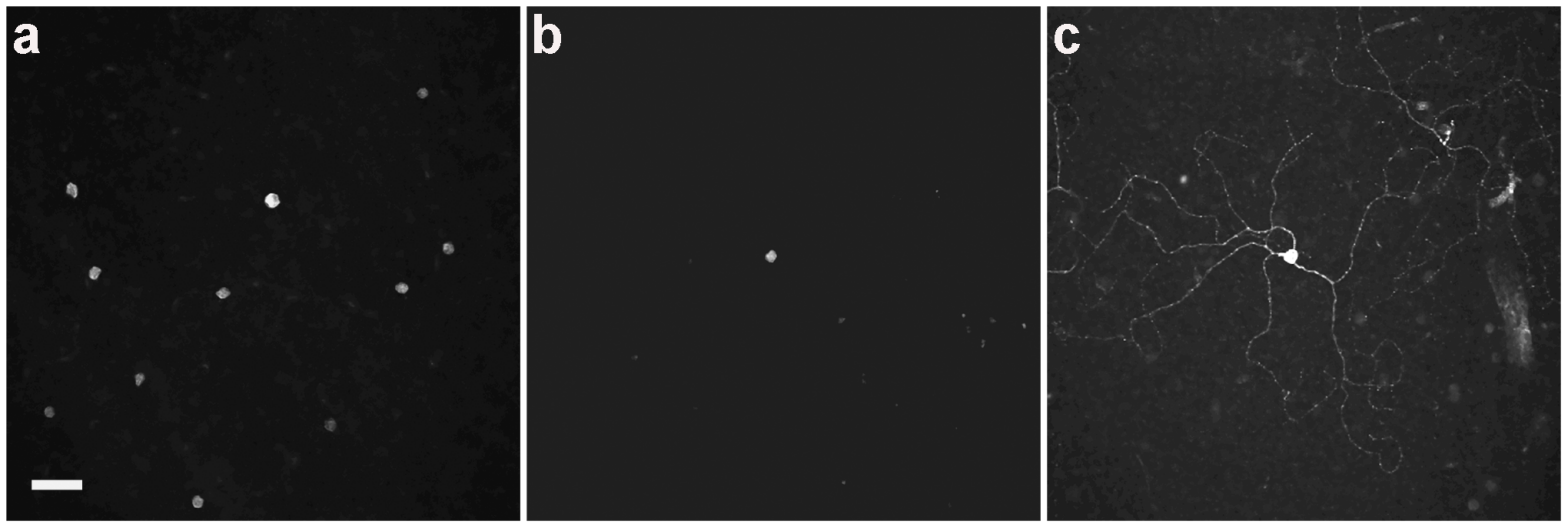
Displaced ganglion cells in the rat retina. (a) RBPMS immunostaining shows numerous displaced ganglion cells in the control eye. (b) The majority of the RBPMS-positive cells are lost 21 days after axotomy. The surviving displaced ganglion cells (b) correspond to M1 ganglion cells (c). Scale bar = 50 μm.

RGCs located in the inner nuclear layer (INL), known as displaced ganglion cells, which are reported to constitute about 2 - 3% of the total RGC population in mouse [22], [23] and rat retina [24], were also investigated. At least 16 different types of displaced ganglion cells have been described in the rodent retina [23]. Figure 5a shows a confocal image of RBPMS immunoreactivity in displaced ganglion cells in the control eye. After the ONT, few RBPMS immunoreactive cells were identified (**Fig. 5b**). Displaced M1 ganglion cells were the only displaced RGCs that were present 21 (n = 3 retinas; **Fig. 5c**) and 60 days following ONT (not shown; n = 3 retinas).

## Discussion

M1 ganglion cells comprise the largest population of RGCs amongst all surviving RGC types following nerve injury; M1 ganglion cells comprised 82±8% of the surviving RGCs two months after ONT. Several groups have reported that some RGCs are more resistant to axotomy, all of which were reported to have large cell bodies [25]–[29].

M1 ganglion cells are reported to be resistant to death in several injury and disease models, including axotomized mouse RGCs and chronic ocular hypertension [28]–[30]. A study performed by Mey and Thanos (1993) described two “types” of RGCs that were resistant to axotomy at 3 weeks. One of the RGCs was described as a “large ganglion cell,” which resembles the ON α-like ganglion cell in our study. The second RGC type was the only cell type that survived one year following axotomy, which was described as a “new type” of RGC characterized by long and meandering dendrites. The descriptions of the morphology and dendritic field size of these cells match that of the M1 ganglion cells.

The minority (1.2±0.3%) of surviving RGCs that were neither M1- nor NF-positive (60 days following ONT) could correspond to another RGC type that also survives axotomy. One possibility is that these cells belong to another melanopsin ganglion cell type. However, to our knowledge, only the M1 ganglion cell type has been reported in rat retina compared to the five different melanopsin ganglion cell types in mouse retina [31]. In view of the reports that utilized genetic techniques and improved antibodies to identify multiple cell types in mouse [31]–[33], there is a possibility that the other surviving cell types also contain low levels of melanopsin, which are below the level of detectability of the antibody used for these studies.

The other RGC that remained after axotomy is an ON α-like ganglion cell, which corresponds best morphologically to M4 melanopsin ganglion cells in the mouse retina [34]. Future experiments are needed to determine whether the surviving ON RGC type is a melanopsin-containing RGC. Experiments could include light response recordings, intrinsic membrane properties and photocurrents to examine whether they exhibit sustained, synaptically driven ON responses as reported for the M4 ganglion cells [34].

We have shown that the surviving M1 ganglion cells undergo changes in their dendritic field size at 7 and 60 days following ONT. Dendritic elongation/reduction might be attributed to the absence of neighboring neurons and dendrites in the IPL. When dendrites are depleted, M1 cell dendrites might have a preference in the direction of their growth in response to the loss of synaptic contacts to ganglion cells, amacrine cells and bipolar cells. Precedents for asymmetric growth in RGC dendrites have been shown after local lesions in the retina [16], [17].

One possible explanation for the survival of M1 ganglion cells is their close relationship to dopaminergic amacrine cells. Since dopamine has been shown to be neuroprotective against glutamate-related neurotoxicity in rat retinal neuron cultures and against glutamate-induced excitotoxicity [35], [36], synaptic contacts between the dendrites of M1 ganglion cells and dopaminergic amacrine cells processes [37] could provide support to the RGCs to prevent degeneration.

A second possibility is that surviving RGCs may be supported by other retinal cells located in the INL that do not contact M1 dendrites. A subpopulation of M1 ganglion cells have collateral axons that do not leave the eye and innervate the IPL [38]. These axonal collaterals could form a novel retinal circuit that maintains M1 ganglion cells after optic nerve injury.

Finally, there are three major survival mechanisms that may impact these cells. A molecular mechanism mediating ganglion cell survival might involve PTEN/mTOR, JAK/STAT or PACAP pathways. The PTEN/mTOR pathway, which regulates dendritic growth in cortical pyramidal neurons [39] and supports ganglion cell axon regeneration in the retina [40], may also play a neuroprotective role in RGCs, including M1 ganglion cells. This speculation follows from studies showing that inhibition of the PTEN/mTOR pathway provides protection against many pathologies including neurodegenerative diseases, cancer, heart diseases, obesity and kidney disease [41]–[43]. Akt plays a critical role in controlling the balance between survival and apoptosis [44]–[46] and is activated by insulin and various growth and survival factors involving phosphatidylinositol 3-kinase (PI3-kinase) [44], [45]. The phosphatase and tensin homologue deleted on chromosome ten (PTEN) is a major suppressor of phosphoinositide-3 kinase/Akt signaling, a vital survival pathway in lung parenchymal cells, RGCs, ischemic brain injury, and traumatic brain injury [47]–[49]. PTEN plays a key role in cell migration, survival and apoptosis by negatively regulating phosphoproteins in the PI3K/Akt pathway.

A second possible mechanism mediating RGC survival is an activation of the janus family tyrosine kinases (JAKs) and signal transducer and activator of transcription (STAT) proteins (JAK/STAT pathway). IL-6 is an activator of the JAK/STAT pathway, which is produced by Müller cells and rescue photoreceptors under stress and RGCs after ischemia-reperfusion in vivo [50], [51]. This pathway is involved in cellular survival, proliferation, differentiation and apoptosis [52].

An alternative mechanism could be mediated by the neuropeptide pituitary adenylate cyclase-activating polypeptide (PACAP), which has a cytoprotective action in neurons and other tissues [53]. Since PACAP is colocalized with the melanopsin-containing ganglion cells [54], it would be interesting to determine whether M1 ganglion cells survive ONT in a PACAP knockout mouse.

Gap junctions are channels that are also involved in allowing the passage of proapoptotic signals between apoptotic cells and healthy surrounding cells [55], [56]. Although it is unclear which molecules are involved in promoting apoptosis, some proapoptotic signal molecules could include Ca^2+^, inositol triphosphate (IP_3_), ATP and cAMP [55]. It has been reported that the effect of survival and apoptosis through gap junctions is partially connexin-dependent [55]–[57]. In the retina, most RGC express Cx36 [58] and a few RGC types express Cx45 [59] and Cx30.2 [60]. It has been postulated that melanopsin RGCs express Cx30.2 [61], which has the lowest single channel conductance among all members of the connexin family [62], and are coupled to amacrine cells rather than ganglion cells [61]. One might speculate that the combination of these factors might impede the passage of death signals from apoptotic ganglion cells to the melanopsin RGCs.

In conclusion, M1 ganglion cells are an optic nerve injury-resistant RGC subtype that offers an exceptional opportunity to explore neuroprotective molecules which could mitigate the loss of vision following optic nerve injury.

## References

[1] Quigley HA, Broman AT (2006) The number of people with glaucoma worldwide in 2010 and 2020. Br J Ophthalmol 90: 262–267. 16488940 10.1136/bjo.2005.081224 PMC1856963

[2] Berkelaar M, Clarke DB, Wang YC, Bray GM, Aguayo AJ (1994) Axotomy results in delayed death and apoptosis of retinal ganglion cells in adult rats. J Neurosci 14: 4368–4374. 8027784 10.1523/JNEUROSCI.14-07-04368.1994 PMC6577016

[3] Robinson GA (1994) Immediate early gene expression in axotomized and regenerating retinal ganglion cells of the adult rat. Brain Res Mol Brain Res 24: 43–54. 7968376 10.1016/0169-328x(94)90116-3

[4] Villegas-Pérez MP, Vidal-Sanz M, Bray GM, Aguayo AJ (1988) Influences of peripheral nerve grafts on the survival and regrowth of axotomized retinal ganglion cells in adult rats. J Neurosci 8: 265–280. 2448429 10.1523/JNEUROSCI.08-01-00265.1988 PMC6569372

[5] Villegas-Pérez MP, Vidal-Sanz M, Rasminsky M, Bray GM, Aguayo AJ (1993) Rapid and protracted phases of retinal ganglion cell loss follow axotomy in the optic nerve of adult rats. J Neurobiol 24: 23–36. 8419522 10.1002/neu.480240103

[6] Cenni MC, Bonfanti L, Martinou JC, Ratto GM, Strettoi E, et al (1996) Long-term survival of retinal ganglion cells following optic nerve section in adult bcl-2 transgenic mice. Eur J Neurosci 8: 1735–1745. 8921264 10.1111/j.1460-9568.1996.tb01317.x

[7] Mey J, Thanos S (1993) Intravitreal injections of neurotrophic factors support the survival of axotomized retinal ganglion cells in adult rats in vivo. Brain Res 602: 304–317. 8448673 10.1016/0006-8993(93)90695-j

[8] Rodriguez A, Pérez de Sevilla L, Brecha N (in press) The RNA binding protein RBPMS is a selective marker of ganglion cells in the mammalian retina. J Comp Neurol. 10.1002/cne.23521 PMC3959221 24318667

[9] Kwong JM, Caprioli J, Piri N (2010) RNA binding protein with multiple splicing: a new marker for retinal ganglion cells. Invest Ophthalmol Vis Sci 51: 1052–1058. 19737887 10.1167/iovs.09-4098 PMC3979483

[10] Drager UC, Hofbauer A (1984) Antibodies to heavy neurofilament subunit detect a subpopulation of damaged ganglion cells in retina. Nature 309: 624–626. 6203041 10.1038/309624a0

[11] Shaw G, Weber K (1984) The intermediate filament complement of the retina: a comparison between different mammalian species. Eur J Cell Biol 33: 95–104. 6538136

[12] Straznicky C, Vickers JC, Gabriel R, Costa M (1992) A neurofilament protein antibody selectively labels a large ganglion cell type in the human retina. Brain Res 582: 123–128. 1498675 10.1016/0006-8993(92)90325-4

[13] Vickers JC, Costa M (1992) The neurofilament triplet is present in distinct subpopulations of neurons in the central nervous system of the guinea-pig. Neurosci 49: 73–100. 10.1016/0306-4522(92)90077-f 1407552

[14] Wang L, Dong J, Cull G, Fortune B, Cioffi GA (2003) Varicosities of intraretinal ganglion cell axons in human and nonhuman primates. Invest Ophthalmol Vis Sci 44: 2–9. 12506048 10.1167/iovs.02-0333

[15] Sun W, Li N, He S (2002) Large-scale morphological survey of rat retinal ganglion cells. Vis Neurosci 19: 483–493. 12511081 10.1017/s0952523802194107

[16] Linden R, Perry VH (1982) Ganglion cell death within the developing retina: a regulatory role for retinal dendrites? Neurosci 7: 2813–2827. 10.1016/0306-4522(82)90104-x 7155355

[17] Perry VH, Linden R (1982) Evidence for dendritic competition in the developing retina. Nature 297: 683–685. 7088156 10.1038/297683a0

[18] Hattar S, Liao HW, Takao M, Berson DM, Yau KW (2002) Melanopsin-containing retinal ganglion cells: Architecture, projections, and intrinsic photosensitivity. Science 295: 1065–1070. 11834834 10.1126/science.1069609 PMC2885915

[19] Baver SB, Pickard GE, Sollars PJ, Pickard GE (2008) Two types of melanopsin retinal ganglion cell differentially innervate the hypothalamic suprachiasmatic nucleus and the olivary pretectal nucleus. Eur J Neurosci 27: 1763–1770. 18371076 10.1111/j.1460-9568.2008.06149.x

[20] Ingham ES, Günhan E, Fuller PM, Fuller CA (2009) Immunotoxin-induced ablation of melanopsin retinal ganglion cells in a non-murine mammalian model. J Comp Neurol 516: 125–140. 19575450 10.1002/cne.22103

[21] Ota T, Hara H, Miyawaki N (2002) Brain-derived neurotrophic factor inhibits changes in soma-size of retinal ganglion cells following optic nerve axotomy in rats. J Ocul Pharmacol Ther 18: 241–249. 12099545 10.1089/108076802760116160

[22] Drager UC, Olsen JF (1981) Ganglion cell distribution in the retina of the mouse. Invest Ophthalmol Vis Sci 20: 285–293. 6162818

[23] Pang JJ, Wu SM (2011) Morphology and immunoreactivity of retrogradely double-labeled ganglion cells in the mouse retina. Invest Ophthalmol Vis Sci 52: 4886–4896. 21482641 10.1167/iovs.10-5921 PMC3175970

[24] Liu ZH, Jen LS (1986) Displaced retinal ganglion cells in normal rats and rats with one eye enucleated at birth. Neurosci Lett 67: 239–244. 3737010 10.1016/0304-3940(86)90315-0

[25] Holländer H, Bisti S, Maffei L, Hebel R (1984) Electroretinographic responses and retrograde changes of retinal morphology after intracranial optic nerve section: a quantitative analysis in the cat. Exp Brain Res 55: 483–493. 6468554 10.1007/BF00235279

[26] Watanabe M, Sawai H, Fukuda Y (1993) Number, distribution, and morphology of retinal ganglion cells with axons regenerated into peripheral nerve grafts in adult cats. J Neurosci 13: 2105–2117. 8478691 10.1523/JNEUROSCI.13-05-02105.1993 PMC6576586

[27] Silveira LC, Russelakis-Carneiro M, Perry VH (1994) The ganglion cell response to optic nerve injury in the cat: differential responses revealed by neurofibrillar staining. J Neurocytol 23: 75–86. 8195813 10.1007/BF01183863

[28] Robinson A, Madison RM (2004) Axotomized mouse retinal ganglion cells containing melanopsin show enhanced survival, but not enhanced axon regrowth into a peripheral nerve graft. Vision Res 44: 2667–2674. 15358062 10.1016/j.visres.2004.06.010

[29] Li RS, Chen BY, Tay DK, Chan HH, Pu ML, et al (2006) Melanopsin-expressing retinal ganglion cells are more injury-resistant in a chronic ocular hypertension model. Invest Ophthalmol Vis Sci 47: 2951–2958. 16799038 10.1167/iovs.05-1295

[30] Deparis S, Caprara C, Grimm C (2012) Intrinsically photosensitive retinal ganglion cells are resistant to N-methyl-D-aspartic acid excitotoxicity. Mol Vis 18: 2814–2827. 23233784 PMC3519378

[31] Ecker JL, Dumitrescu ON, Wong KY, Alam NM, Chen SK, et al (2010) Melanopsin-expressing retinal ganglion-cell photoreceptors: cellular diversity and role in pattern vision. Neuron 67: 49–60. 20624591 10.1016/j.neuron.2010.05.023 PMC2904318

[32] Do MT, Kang SH, Xue T, Zhong H, Liao HW, et al (2008) Photon capture and signalling by melanopsin retinal ganglion cells. Nature 457: 281–7. 19118382 10.1038/nature07682 PMC2794210

[33] Schmidt TM, Taniguchi K, Kofuji P (2008) Intrinsic and extrinsic light responses in melanopsin-expressing ganglion cells during mouse development. J Neurophysiol 100: 371–384. 18480363 10.1152/jn.00062.2008 PMC2493479

[34] Estevez ME, Fogerson PM, Ilardi MC, Borghuis BG, Chan E, et al (2012) Form and function of the M4 cell, an intrinsically photosensitive retinal ganglion cell type contributing to geniculocortical vision. J Neurosci 32: 13608–13620. 23015450 10.1523/JNEUROSCI.1422-12.2012 PMC3474539

[35] Kitaoka Y, Kumai T (2004) Modulation of retinal dopaminergic cells by nitric oxide. A protective effect on NMDA-induced retinal injury. In Vivo 18: 311–315. 15341186

[36] Vaarmann A, Kovac S, Holmström KM, Gandhi S, Abramov AY (2013) Dopamine protects neurons against glutamate-induced excitotoxicity. Cell Death Dis 4: e455. 23303129 10.1038/cddis.2012.194 PMC3563982

[37] Vugler AA, Redgrave P, Semo M, Lawrence J, Greenwood J, et al (2007) Dopamine neurones form a discrete plexus with melanopsin cells in normal and degenerating retina. Exp Neurol 205: 26–35. 17362933 10.1016/j.expneurol.2007.01.032

[38] Joo HR, Peterson BB, Dacey DM, Hattar S, Chen SK (2013) Recurrent axon collaterals of intrinsically photosensitive retinal ganglion cells. Vis Neurosci 9: 1–8. 10.1017/S0952523813000199 PMC4316817 23834959

[39] Chow DK, Groszer M, Pribadi M, Machniki M, Carmichael ST, et al (2009) Laminar and compartmental regulation of dendritic growth in mature cortex. Nat Neurosci 12: 116–118. 19151711 10.1038/nn.2255 PMC2842592

[40] de Lima S, Koriyama Y, Kurimoto T, Oliveira JT, Yin Y, et al (2012) Full-length axon regeneration in the adult mouse optic nerve and partial recovery of simple visual behaviors. Proc Natl Acad Sci USA 109: 9149–9154. 22615390 10.1073/pnas.1119449109 PMC3384191

[41] Blanco-Aparicio C, Renner O, Leal JF, Carnero A (2007) PTEN, more than the AKT pathway. Carcinogenesis 28: 1379–1386. 17341655 10.1093/carcin/bgm052

[42] Sun F, Park KK, Belin S, Wang D, Lu T, et al (2011) Sustained axon regeneration induced by co-deletion of PTEN and SOCS3. Nature Letters 480: 372–375. 10.1038/nature10594 PMC3240702 22056987

[43] Johnson CS, Rabinovitch SP, Kaeberlein M (2013) mTOR is a key modulator of ageing and age-related disease. Nature 493: 338–345. 23325216 10.1038/nature11861 PMC3687363

[44] Burgering BM, Coffer PJ (1995) Protein kinase B (c-Akt) in phosphatidylinositol-3-OH kinase signal transduction. Nature 376: 599–602. 7637810 10.1038/376599a0

[45] Franke TF, Yang SI, Chan TO, Datta K, Kazlauskas A, et al (1995) The protein kinase encoded by the Akt proto-oncogene is a target of the PDGF-activated phosphatidylinositol 3-kinase. Cell 81: 727–36. 7774014 10.1016/0092-8674(95)90534-0

[46] Franke TF, Kaplan DR, Cantley LC (1997) PI3K: downstream AKTion blocks apoptosis. Cell 88: 435–437. 9038334 10.1016/s0092-8674(00)81883-8

[47] Noshita N, Lewén A, Sugawara T, Chan PH (2002) Akt phosphorylation and neuronal survival after traumatic brain injury in mice. Neurobiol Dis 9: 294–304. 11950275 10.1006/nbdi.2002.0482

[48] Li SY, Yau SY, Chen BY, Tay DK, Lee VW, et al (2008) Enhanced survival of melanopsin-expressing retinal ganglion cells after injury is associated with the PI3 K/Akt pathway. Cell Mol Neurobiol 28: 1095–1107. 18512147 10.1007/s10571-008-9286-x PMC11514987

[49] Lai JP, Bao S, Davis IC, Knoell DL (2009) Inhibition of the phosphatase PTEN protects mice against oleic acid-induced acute lung injury. Br J Pharmacol 156: 189–200. 19134000 10.1111/j.1476-5381.2008.00020.x PMC2697782

[50] Joly S, Lange C, Thiersch M, Samardzija M, Grimm C (2008) Leukemia inhibitory factor extends the lifespan of injured photoreceptors in vivo. J Neurosci 28: 13765–13774. 19091967 10.1523/JNEUROSCI.5114-08.2008 PMC6671917

[51] Zhang C, Li H, Liu MG, Kawasaki A, Fu XY, et al (2008) STAT3 activation protects retinal ganglion cell layer neurons in response to stress. Exp Eye Res 86: 991–997. 18471811 10.1016/j.exer.2008.03.020

[52] Rane SG, Reddy EP (2000) Janus kinases: components of multiple signaling pathways. Oncogene 19: 5662–5679. Review. 10.1038/sj.onc.1203925 11114747

[53] Reglodi D, Kiss P, Szabadfi K, Atlasz T, Gabriel R, et al (2012) PACAP is an endogenous protective factor-insights from PACAP-deficient mice. J Mol Neurosci 48: 482–492. 22528455 10.1007/s12031-012-9762-0

[54] Hannibal J, Hindersson P, Ostergaard J, Georg B, Heegaard S, et al (2004) Melanopsin is expressed in PACAP-containing retinal ganglion cells of the human retinohypothalamic tract. Invest Ophthalmol Vis Sci. 45: 4202–4209. 10.1167/iovs.04-0313 15505076

[55] Krysko DV, Leybaert L, Vandenabeele P, D'Herde K (2005) Gap junctions and the propagation of cell survival and cell death signals. Apoptosis. 10: 459–469. Review. 10.1007/s10495-005-1875-2 15909108

[56] Rodríguez-Sinovas A, Cabestrero A, López D, Torre I, Morente M, et al. (2007) The modulatory effects of connexin 43 on cell death/survival beyond cell coupling. Prog Biophys Mol Biol 94: 219–232. Review. 10.1016/j.pbiomolbio.2007.03.003 17462722

[57] Kameritsch P, Khandoga N, Pohl U, Pogoda K (2013) Gap junctional communication promotes apoptosis in a connexin-type-dependent manner. Cell Death Dis 4: e584. 23579271 10.1038/cddis.2013.105 PMC3641328

[58] Pan F, Paul DL, Bloomfield SA, Völgyi B (2010) Connexin36 is required for gap junctional coupling of most ganglion cell subtypes in the mouse retina. J Comp Neurol 518: 911–927. 20058323 10.1002/cne.22254 PMC2860380

[59] Schubert T, Maxeiner S, Krüger O, Willecke K, Weiler R (2005) Connexin45 mediates gap junctional coupling of bistratified ganglion cells in the mouse retina. J Comp Neurol 490: 29–39. 16041717 10.1002/cne.20621

[60] Pérez de Sevilla Müller L, Dedek K, Janssen-Bienhold U, Meyer A, Kreuzberg MM, et al (2010a) Expression and modulation of connexin 30.2, a novel gap junction protein in the mouse retina. Vis Neurosci 27: 91–101. 20537217 10.1017/S0952523810000131

[61] Pérez de Sevilla Müller L, Do MT, Yau KW, He S, Baldridge WH (2010b) Tracer coupling of intrinsically photosensitive retinal ganglion cells to amacrine cells in the mouse retina. J Comp Neurol 518: 4813–4824. 20963830 10.1002/cne.22490 PMC2967574

[62] Kreuzberg MM, Schrickel JW, Ghanem A, Kim JS, Degen J, et al (2006) Connexin30.2 containing gap junction channels decelerate impulse propagation through the atrioventricular node. Proc Natl Acad Sci U S A (103) 5959–5964. 10.1073/pnas.0508512103 PMC1458680 16571663

